# Evidence for pharmacological interventions to reduce cardiovascular risk for patients with chronic kidney disease: a study protocol of an evidence map

**DOI:** 10.1186/s13643-022-02108-x

**Published:** 2022-11-12

**Authors:** Julia M. T. Colombijn, Demy L. Idema, Kim van der Braak, Rene Spijker, Sabine C. A. Meijvis, Michiel L. Bots, Lotty Hooft, Marianne C. Verhaar, Robin W. M. Vernooij

**Affiliations:** 1 Department of Nephrology & Hypertension, University Medical Centre Utrecht, Heidelberglaan 100, 3584 CX Utrecht, the Netherlands; 2 Julius Centre for Health Sciences and Primary Care, University Medical Centre Utrecht, Utrecht University, Utrecht, the Netherlands; 3 Cochrane Netherlands, Julius Center for Health Sciences and Primary Care, University Medical Center Utrecht, Utrecht University, Utrecht, the Netherlands; 4 Medical Library, Amsterdam UMC, University of Amsterdam, Amsterdam Public Health, Amsterdam, the Netherlands

**Keywords:** Chronic kidney disease, Cardiovascular disease, Cardiovascular risk management, Evidence map

## Abstract

**Background:**

Patients with chronic kidney disease (CKD) require a personalised strategy for cardiovascular risk management (CVRM) to reduce their high risk of cardiovascular morbidity and mortality. Despite their high risk, patients with CKD appear to be underrepresented in randomised controlled trials (RCTs) for pharmacological CVRM interventions to reduce cardiovascular risk (pharmacological CVRM interventions). As a result, it remains unclear whether the efficacy of these interventions found in patients without CKD is similarly applicable to patients with CKD. This evidence map aims to provide an overview of the availability of the evidence from pharmacological CVRM trials for patients with CKD by assessing how often patients with reduced kidney function are specifically excluded or included from RCTs on pharmacological CVRM interventions and whether studies report efficacy estimates of interventions specifically for kidney patients.

**Methods:**

We will perform a systematic literature search in ClinicalTrials.gov to identify relevant planned, ongoing, and completed RCTs on a broad range of CVRM medications after which we will retrieve the published protocols and papers via ClinicalTrials.gov itself, Embase, MEDLINE, or Google Scholar. We will include RCTs that investigate the efficacy of platelet inhibitors, anticoagulants, antihypertensives, glucose-lowering medication, and lipid-lowering medication on all-cause mortality, cardiovascular mortality, cardiovascular morbidity, and end-stage kidney disease in patients with a cardiovascular history or a major risk factor for cardiovascular disease. Two reviewers will independently screen trial records and their corresponding full-text publications to determine eligibility and extract data. Outcomes of interest are the exclusion of patients with reduced kidney function from RCTs and whether the study population was restricted to kidney patients or subgroup analyses were performed on kidney function. Results will be visualised in an evidence map.

**Discussion:**

The availability of evidence on the efficacy and safety of pharmacological CVRM interventions in patients with CKD might be limited. Hence, we will identify knowledge gaps for future research. At the same time, the availability of evidence, or lack thereof, might warrant caution from healthcare decision-makers in making strong recommendations based on the extrapolation of results from studies to patients who were explicitly excluded from participation.

**Systematic review registration:**

PROSPERO CRD42022296746.

**Supplementary Information:**

The online version contains supplementary material available at 10.1186/s13643-022-02108-x.

## Background

Chronic kidney disease (CKD) is highly prevalent accounting for 1.2 million deaths globally every year [1]. Patients with CKD are at high risk for cardiovascular disease (CVD) independent of diabetes or hypertension [2, 3]. CVD is the main cause of death for patients with CKD stages 3 to 5 (estimated glomerular filtration rate (eGFR) < 60 ml/min/1.73m^2^) [3]. CVD risk already increases in patients with an eGFR of 75 ml/min/1.73m^2^ or lower [4, 5]. In fact, the vast majority of patients with CKD are at greater risk for CVD and cardiovascular mortality than for progressing to end-stage kidney disease (ESKD) [6, 7].

The high risk of CVD in patients with CKD makes cardiovascular risk management (CVRM) indispensable for them. In order to provide personalised CVRM treatment for patients with CKD, the efficacy of pharmacological CVRM interventions should be determined specifically for this patient population. Patients with CKD are a very heterogeneous patient population in terms of age, comorbidities, CKD stage, and cause of kidney failure and have competing risks for mortality and ESKD [8–10]. Consequently, the effects of interventions may differ for patients with and without CKD or for patients with different stages of CKD.

Evidence for the efficacy of pharmacological CVRM interventions in patients with CKD is likely to be scant. Even if patients with CKD are included in randomised controlled trials (RCTs), authors do not necessarily investigate whether the treatment effects differ between patients with normal and reduced kidney function. Results from four reviews including RCTs published between 1985 and 2014 suggest that patients with CKD are systematically underrepresented in clinical trials on pharmacological CVRM interventions [11–14]. Since then, the CVRM landscape has vastly changed with the introduction of, for example, direct oral anticoagulants (DOACs), proprotein convertase subtilisin/kexin type 9 (PCSK-9) inhibitors, and sodium-glucose co-transporter-2 (SGLT-2) inhibitors. The previous reviews are also likely to have missed studies published in smaller journals, since their search was predominantly limited to the major general medicine, cardiology, and nephrology journals. In addition, they restricted themselves to RCTs investigating the efficacy of a selection of antihypertensives, antiplatelets, anticoagulants, and statins on all-cause mortality and coronary artery disease in patients with pre-established CVD.

Despite the urgency for personalised CVRM for patients with CKD, no review has investigated for which pharmacological CVRM interventions the efficacy for patients with reduced kidney function has been determined in RCTs. Therefore, we will create an evidence map to provide an overview of the available evidence for pharmacological CVRM interventions on cardiovascular and kidney endpoints for patients with CKD. We will explore how often patients with reduced kidney function are excluded from RCTs on pharmacological CVRM interventions and whether the authors restricted the study population to patients with reduced kidney function or performed subgroup analysis to determine the effect in patients with reduced kidney function.

## Methods/design

The protocol is registered in the International Prospective Register for Systematic Reviews (PROSPERO CRD42022296746) and was written based on the Preferred Reporting Items for Systematic Reviews and Meta-Analysis Protocols (PRISMA-P) [15].

### Eligibility criteria

#### Study design

We will include only RCTs. Reviews, meta-analyses, observational studies, case reports, animal studies, and cross-over trials will be excluded. No restrictions will be applied on language and publication status.

#### Population

We will include RCTs whose study population consists of adults with a history of CVD or adults without a history of CVD with at least one cardiovascular risk factor. History of CVD will be defined as the presence of coronary artery disease, atrial fibrillation, congestive heart failure, peripheral arterial disease, stroke, or aortic aneurysm. Patients with one or more of the following cardiovascular risk factors will also be included: overweight or obesity, hypertension, hyperglycaemia or diabetes mellitus, or chronic- or end-stage kidney disease (Table 1) [3, 16, 17]. Studies with a sample size < 100 and studies in paediatric patients will be excluded. Studies in both paediatric and non-paediatric patients will be included if stratified analyses were performed for non-paediatric patients.

**Table 1:** Definitions of included cardiovascular risk factors

Risk factor	definition
Obesity	• BMI > 25 kg/m^2^ • Waist circumference ≥ 88 cm (W) or ≥ 102 cm (M)
Hypertension	• Blood pressure ≥ 130/85 mmHg • Prescription of antihypertensives • Hypertension as defined by authors
Dyslipidaemia	• History of familial hypercholesterolaemia or hyperlipidaemia • Triglycerides > 150 mg/dL (> 1.7 mmol/L) • HDL cholesterol < 40 mg/dL (< 1.03 mmol/L) (M) or < 50 mg/dL (< 1.29 mmol/L) (W) • Prescription of lipid-lowering medication
Hyperglycaemia/diabetes	• Fasting glucose ≥ 110 mg/dL (≥ 6.1 mmol/L) • Prescription of glucose-lowering medication • Diabetes or pre-diabetes as defined by authors
Chronic kidney disease	• eGFR < 60 ml/min/1.73m^2^ • Albuminuria (AER ≥ 30 mg/24 h; ACR ≥ 30 mg/g ≥ 3 mg/mmol) • Dialysis • Chronic kidney disease or end-stage kidney disease as defined by authors

*Abbreviations: BMI* body mass index, *W* women, *M* men, *HDL* high-density cholesterol *eGFR* estimated glomerular filtration rate, *AER* albumin excretion rate, *ACR* albumin creatinine ratio

#### Interventions and comparators

We will include studies that examine pharmacological interventions recommended by the European Society of Cardiology (ESC), the American Heart Association (AHA), the American Stroke Association (ASA), the American College of Cardiology (ACC), or the American Diabetes Association (ADA) for the prevention of cardiovascular disease in general and treatment of cardiovascular risk factors [18–39]. Broadly speaking, these concern antihypertensives, cholesterol-lowering medication, glucose-lowering medication, anticoagulants, or anti-platelets (for a full list, see Table 2). Interventions must be compared against placebo, no treatment, usual care, another therapy, or a different dosage or duration of treatment. Studies that investigated a combination of pharmacological and non-pharmacological interventions (e.g., coronary artery bypass graft (CABG) + antiplatelet therapy vs CABG only) will be included. Studies in which the intervention is first administered in an acute setting or during a cardiac or vascular procedure will be included if the intervention is continued during the follow-up. Studies that compare different treatment targets (e.g. systolic blood pressure < 140 mmHg vs < 130 mmHg) will be excluded.

**Table 2:** Overview of eligible medication classes included

Antihypertensives	Lipid-lowering medications	Anti-platelets and anticoagulants	Glucose-lowering medications
1. Alpha-blockers	1. HMG-CoA inhibitors	1. Salycates	1. Biguanides
2. ACE-inhibitors	2. Fibrates	2. P2Y12 inhibitors	2. Sulfonylureas
3. Angiotensin receptor blockers	3. PCSK-9 inhibitors	3. GP IIb/IIIa inhibitors	3. DPP-4 inhibitors
4. Aldosterone antagonist	4. Niacin/nicotinic acid	4. Other platelet inhibitors	4. GLP-1 receptor agonists
5. Renin inhibitors	5. Bile acid sequestrants	5. Heparins	5. SGLT-2 inhibitors
6. Beta-blockers	6. Selective cholesterol absorption inhibitors	6. Vitamin K antagonists	6. Alpha-glucosidase inhibitors
7. Dihydropyridine calcium channel blockers	7. Other lipid-lowering agents	7. Direct oral anti-coagulants	7. Thiazolidinediones
8. Non-dihydropyridine calcium channel blockers			8. Meglitinides
9. Thiazide(-like) diuretics			9. Insulin
10. Loop diuretics			
11. Vasodilators			
12. Centrally acting antihypertensives			

Other lipid-lowering medications: probucol, policosanol, mipomersen, lomitapide, bempedoic acid, and icosapent ethyl

Other antiplatelet medications: dipyridamole, picotamide, ticlopidine, indobufen, iloprost, triflusal, cilostazol, and vorapaxar

*Abbreviations: ACE* angiotensin-converting enzyme, *HMG-CoA* β-hydroxy β-methylglutaryl co-enzyme A, *PCSK-9* proprotein convertase subtilisin/kexin type 9, *GP IIb/IIIa* glycoprotein IIb/IIIa, *DPP-4* dipeptidyl-peptidase 4, *GLP-1* glucagonlike peptide-1, *SGLT-2* sodium-glucose cotransporter-2

#### Outcomes

We will restrict ourselves to studies which report patient-important outcomes since these are most likely to drive clinical decision-making [40–42]. Included studies must report at least one of the following endpoints: all-cause mortality, cardiovascular mortality, major cardiovascular event (MACE) or another composite cardiovascular endpoint, coronary artery disease (myocardial infarction, angina, coronary artery bypass grafting, percutaneous coronary intervention, or as defined by authors), cerebrovascular disease (stroke, transient ischaemic attack, or as defined by authors), peripheral arterial disease (bypass, amputation, aortic aneurysm, or as defined by authors), hospitalisation for heart failure or development of heart failure stage III or IV (New York Heart Association classification), or ESKD (initiation of dialysis, kidney transplant, or as defined by authors). Studies reporting only surrogate endpoints will be excluded.

### Search and study selection

Our search strategy will consist of a two-step approach. First, we will perform a search in Cochrane Central to identify relevant planned, ongoing, and completed RCTs in the ClincialTrials.gov trial registry using a combination of keywords for cardiovascular disease and the interventions of interest (Appendix 1). After identifying relevant RCTs in ClinicalTrials.gov, we will retrieve the publications related to the trial record in ClinicalTrials.gov. If no publications are listed in ClinicalTrials.gov, we will perform an additional search on clinical trial record number (NCT number) and study acronym in Embase, MEDLINE, and Google Scholar. If no publications can be retrieved, the record will be excluded.

Conducting a literature search through a trial registry is a relatively new approach. Since 2005, it is mandatory to register new RCTs in a trial registry [43]. Consequently, it should be possible to identify relevant RCTs through such a trial registry. As a substudy, we will validate our search strategy to identify relevant RCTs from a trial registry by comparing the Clinicaltrials.gov search results with the search results from a search in MEDLINE, Embase, and Google Scholar (Appendix 2). Additionally, we will evaluate selective outcome reporting by comparing the outcomes reported in the trial records and those reported in the study publications and publication bias from the number of studies that have published a protocol (in ClinicalTrials.gov or as separate publication) but have never published results or vice versa. For this validation study, we will limit ourselves to RCT anticoagulants conducted in the past 10 years (2012 to 2022). For the validation search, we will use a comparable set of keywords and MeSH terms as for the search in ClinicalTrials.gov and apply a RCT filter and publication date filter (between 2011 and 2021) [44].

### Screening and data collection

**Fig. 1:**
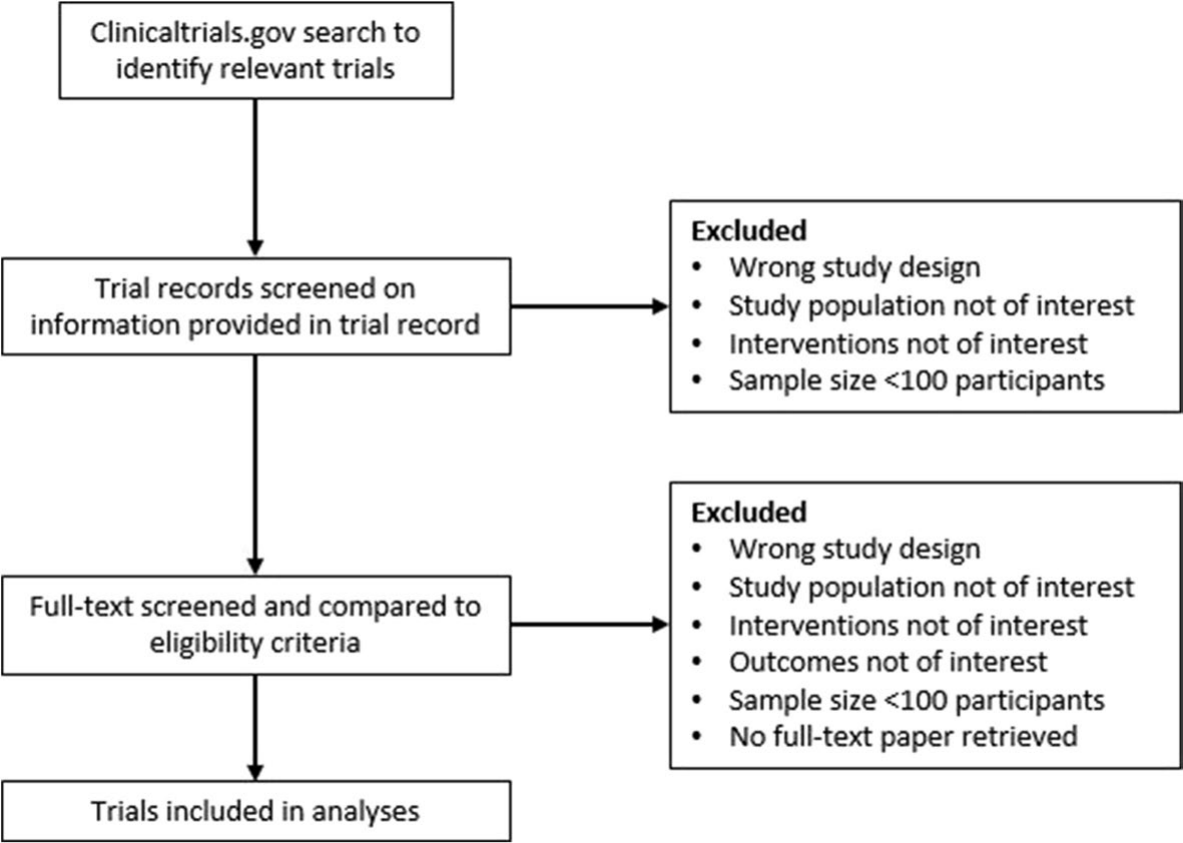
Flowchart of screening and selection process of trials

First, we will screen trial records on title and trial description to select RCTs for full-text screening. Next, we will screen the retrieved full-text articles linked to the trial record to determine if RCTs meet the inclusion criteria. Pairs of reviewers will work independently for both the screening of the trial records and the full-text papers. Disagreements between reviewers on eligibility will be resolved by consensus or by means of an adjudicator. The whole screening process is visualised in Fig. 1.

Pairs of reviewers will independently extract data on patient characteristics such as age, sex, and kidney function with standardised data extraction forms and study characteristics such as study design, reported endpoints, and exclusion of patients on hypertension, pre-diabetes, or smoking (for a full list, see Table 3). For the methodological substudy, we will extract whether results have been published and compare reported outcomes from ClinicalTrials.gov and the published articles.

**Table 3:** Study and patient characteristics extracted from included studies

**Study characteristics**		
Name of trial	Follow-up	Subgroup analysis on kidney function or disease
NCT number	Sample size	Restriction of study population to patients with reduced kidney function
Region	Type of intervention	Hypertension, blood pressure, or use of antihypertensive exclusion criterion^b^
Year of publication	Reported outcomes	Diabetes, HbA1c, or use of anti-glycaemic agent exclusion criterion^b^
Funding source	Kidney function or disease exclusion criterion^a,b^	Smoking exclusion criterion^b^
**Patient characteristics**		
Age		
Sex	Undergoing dialysis	
Baseline kidney function		

^a^Defined as exclusion criterion based on eGFR, serum creatinine, kidney disease, renal insufficiency, end-stage kidney disease, dialysis, or ambiguous kidney exclusion criterion

^b^If not specifically specified, presume that the respective group of patients is included in the trial

The two outcomes of interest are (1) the exclusion of patients with reduced kidney function from RCTs and (2) subgroup analyses on kidney function or restriction of the study population to patients with reduced kidney function. Exclusion of patients with CKD will be extracted according to the definition of the authors of the included studies and categorised into one of the following categories: (e)GFR or creatinine clearance, serum creatinine, kidney insufficiency, pre-established kidney disease, end-stage kidney disease or maintenance dialysis, or an ambiguous kidney-related exclusion criterion. If authors do not explicitly state kidney function or kidney disease as exclusion criterion, we will presume that these patients were not excluded. We will compare the rate of exclusion from RCTs on pharmacological CVRM interventions on CKD with the exclusion on smoking, diabetes, and hypertension, which are other major risk factors for CVD. Subgroup analysis based on kidney function will be defined as performing an analysis based on one exclusion criteria for kidney disease described above.

### Risk of bias assessment

We will not perform a risk of bias assessment because bias in study design will not compromise the data synthesis and conclusions of our study.

### Data synthesis

The data will be summarised descriptively, based on the mean ± standard deviation (SD) and median [interquartile range, IQR] for continuous variables and frequency (percentage) for categorical variables. Results will be presented narratively and in tables and figures. We will report the proportion of studies that exclude patients with reduced kidney function, smoking, diabetes, and hypertension, and the proportion of studies that perform subgroup analyses on kidney function. We will provide an overview of kidney-related exclusion criteria of studies that exclude patients with CKD. Characteristics of studies that include or exclude patients with reduced kidney function, perform subgroup analyses, and report kidney function at baseline will be compared using chi-square tests, independent *t*-tests or non-parametric tests. For the assessment of subgroup analyses and restriction of the study population to patients with reduced kidney function, we will create a visual overview of the available studies with an evidence map in which studies will be clustered on CKD stage, serum creatinine levels, dialysis, or kidney transplant, intervention, and reported outcomes. No meta-analyses of clinical outcomes (e.g. morbidity and mortality) will be performed.

For our methodological substudy, we will match the search results from ClinicalTrials.gov with the search results from a bibliographic database search and report the proportion of matches identified. We will estimate publication bias by the number of studies that have a published protocol (in ClinicalTrials.gov) but which have never published results. Studies that were expected to be finished after 1 January 2019 will not be classified under reporting bias because they might still publish results. Vice versa, as an indication of good research practice, we will look at the proportion of publications of RCTs that do not have a registration in ClinicalTrials.gov. Additionally, for studies that have both a ClinicalTrials.gov registration and a publication of the trial results, we will compare the outcomes that were stated in the registration to the outcomes that were reported in the publication.

### Ethical considerations and dissemination of results

This study does not fall under the Dutch Medical Research Involving Human Subjects Act as it does not involve patients or individual patient data. Therefore, no ethical approval was sought from a medical ethics committee. We intend to disseminate the results of our study through peer-reviewed scientific journals, conferences, and meetings with patient organisations. Since CVRM for CKD patients does not only concern nephrologists but also other medical disciplines like general practitioners, cardiologists, endocrinologists, and vascular medicine specialists, we will target journals that have a broader audience.

## Discussion

Although CVRM is of paramount importance for CKD patients, uncertainty remains about the availability of evidence on pharmacological CVRM interventions specifically for this patient population. This study will provide an overview of how often patients with CKD are excluded from cardiovascular RCTs. Looking at subgroup analyses on kidney function and studies restricted to patients with CKD will allow us to create an overview of what evidence exists for different interventions for specific groups of CKD patients. Hence, we will identify whether an evidence gap exists for certain interventions for this patient population. We will not determine the efficacy of specific interventions due to the large number of expected RCTs, the expected heterogeneity in study designs, and the fact that the efficacy of interventions lies outside the scope of our research objective.

We have taken several steps to increase the relevance of our results for clinical practice. We have limited ourselves to RCTs as these are generally considered to provide the highest level of evidence to determine the effectiveness of such interventions [45, 46] Contrary to previous reviews, we have opted to include both patients with and without pre-established CVD which ensures that we cover the whole spectrum of patients with CKD. We will include the interventions currently recommended in international guidelines as these are most likely prescribed in today’s clinical practice and include a broad set of patient-important outcomes to cover the whole spectrum of preventive indications. We deliberately will not include studies that only report surrogate endpoints such as change in blood pressure, HbA1c, or cholesterol since these outcomes are less relevant to patients and less likely to guide clinical decision-making [40, 41].

Our substudy will have both methodological and clinical implications. A trial registry search may be more efficient means to identify relevant RCTs for a literature search than a search in traditional bibliographic databases. RCTs frequently publish more than one article. Instead of having to screen all the individual articles, one might be able to limit the screening process to the information provided in the trial registry. Validating our search in ClinicalTrials.gov with a bibliographic database search will provide insight into the comprehensiveness of our search results and the viability of performing a literature search in a trial registry. Our substudy will also give an indication of the extensiveness of publication bias in RCTs on CVRM interventions and accuracy of reporting of study protocols in trial registries.

This study will likely have some limitations. We might miss some RCTs when the publications are not linked to the trial record or not registered in the trial registry that we searched. We expect this will include most RCTs conducted before February 2000, when ClinicalTrials.gov became available [47]. We also expect to miss some of the RCTs between February 2000 and September 2007 because in that period registration of a clinical trial in ClinicalTrials.gov was optional [47]. Especially in a fast-pacing field like CVRM, clinical decision-making is largely based on recent trials. Therefore, we will be able to give insight into the current evidence for pharmacological interventions for patients with reduced kidney function. Finally, we will not perform a formal risk of bias assessment since none of our outcomes of interest is related to the methodological aspects usually assessed in a risk of bias assessment.

## Supplementary Information

**Additional file 1: Appendix 1.** search strategy in ClinicalTrials.gov. **Appendix 2.** search strategy for validation study.

## Data Availability

Not applicable.

## Abbreviations

ACC: American College of Cardiology
ADA: American Diabetes Association
AHA: American Heart Association
ASA: American Stroke Association
CABG: Coronary artery bypass graft
CKD: Chronic kidney disease
CVD: Cardiovascular disease
CVRM: Cardiovascular risk management
DOAC: Direct oral anticoagulant
eGFR: Estimated glomerular filtration rate
ESC: European Society of Cardiology
ESKD: End-stage kidney disease
IQR: Interquartile range
MACE: Major cardiovascular event
MeSH: Medical Subject Headings
PCSK-9: Proprotein convertase subtilisin–kexin type 9
PRISMA: Preferred Reporting Items for Systematic Reviews and Meta-Analysis Protocols
PROSPERO: Prospective Register for Systematic Reviews
RCT: Randomised controlled trial
SD: Standard deviation
SGLT-2: Sodium-glucose cotransporter-2
