# Supplementary material for: Evidence for pharmacological interventions to reduce cardiovascular risk for patients with chronic kidney disease: a study protocol of an evidence map
Source: Syst Rev. 2022 Nov 12;11:238. doi: 10.1186/s13643-022-02108-x (PMC9655868; doi:10.1186/s13643-022-02108-x)
Supplement: Supplementary file 1 — Additional file 1: Appendix 1. search strategy in ClinicalTrials.gov. Appendix 2. search strategy for validation study. [file 13643_2022_2108_MOESM1_ESM.docx]

**Appendix 1: search strategy in ClinicalTrials.gov**

| **Included interventions** | |
| --- | --- |
| #1 | ("simvastatin" OR "lovastatin" OR "pravastatin" OR "fluvastatin" OR "atorvastatin" OR "cerivastatin" OR "rosuvastatin" OR "pitavastatin" OR "Clofibrate" OR "Bezafibrate" OR "Gemfibrozil" OR "Ciprofibrate" OR "Etofibrate" OR "Choline fenofibrate" OR "alirocumab" OR "evolocumab" OR "inclisiran" OR "ezetimibe" OR "eicosapentaenoic acid" OR "docosahexaenoic acid" OR "mipomersen" OR "lomitapide" OR "Bempedoic acid" OR "colestyramine" OR "colesevelam" OR "acipimox" OR "niacin" OR "nicotinic acid" OR "benazepril" OR "captopril" OR "cilazapril" OR "delapril" OR "enalapril" OR "fosinopril" OR "lisinopril" OR "moexipril" OR "perindopril" OR "quinapril" OR "ramipril" OR "spirapril" OR "temocapril" OR "trandolapril" OR "zofenopril" OR "Azilsartan" OR "Candesartan" OR "Eprosartan" OR "Fimasartan" OR "Irbesartan" OR "Losartan" OR "Olmesartan" OR "Telmisartan" OR "Valsartan" OR "Aliskiren" OR "remikiren" OR "acebutolol" OR "atenolol" OR "betaxolol" OR "bevantolol" OR "bisoprolol" OR "bopindolol" OR "carvedilol" OR "celiprolol" OR "epanolol" OR "labetalol" OR "metoprolol" OR "nadolol" OR "nebivolol" OR "oxprenolol" OR "penbutolol" OR "pindolol" OR "propanolol" OR "amlodipine" OR "barnidipine" OR "benidipine" OR "felodipine" OR "isradipine" OR "lacidipine" OR "lercanidipine" OR "manidipine" OR "nifedipine" OR "nilvadipine" OR "nisoldipine" OR "nitrendipine" OR "nicardipine" OR "diltiazem" OR "gallopamil" OR "verapamil" OR "mibefradil" OR "bendroflumethiazide" OR "chlorothiazide" OR "chorthalidone" OR "clopamide" OR "cyclopenthiazide" OR "hydrochlorothiazide" OR "hydroflumethiazide" OR "indapamide" OR "mefruside" OR "methyclothiazide" OR "metolazone" OR "trichlormethiazide" OR "xipamide" OR "bumetanide" OR "furosemide" OR "torsemide" OR "piretanide" OR "amiloride" OR "triamterene" OR "spironolactone" OR "eplerenone" OR "canrenone" OR "doxazosin" OR "indoramin" OR "prazosin" OR "terazosin" OR "trimazosin" OR "clonidine" OR "methyldopa" OR "guanfacine" OR "moxonidine" OR "rilmenidine" OR "hydralazine" OR "minoxidil" OR "metformin" OR "repaglinide" OR "gliclazide" OR "glibenclamide" OR "glimepiride" OR "tolbutamide" OR "glipizide" OR "gliquidone" OR "pioglitazone" OR "rosiglitazone" OR "troglitazone" OR "lobeglitazone" OR "acarbose" OR "miglitol" OR "voglibose" OR "repaglinide" OR "nateglinide" OR "pramlintide" OR "benfluorex" OR "mitiglinide" OR "insulin" OR "insulin lispro" OR "insulin aspart" OR "insulin glulisine" OR "insulin degludec" OR "insuline glargine" OR "insulin detemir" OR "insulin isophane" OR "linagliptin" OR "saxagliptin" OR "sitagliptin" OR "vildagliptin" OR "alogliptin" OR "gemigliptin" OR "evogliptin" OR "teneligliptin" OR "dulaglutide" OR "exenatide" OR "liraglutide" OR "lixisenatide" OR "semaglutide" OR "albiglutide" OR "beinaglutide" OR "dapagliflozin" OR "canagliflozin" OR "empagliflozin" OR "ertugliflozin" OR "ipragliflozin" OR "sotagliflozin" OR "luseogliflozin" OR "acetyl salicylic acid" OR"asperin" OR "carbasalate calcium" OR "clopidogrel" OR "prasugrel" OR "ticagrelor" OR "cangrelor" OR "abciximab" OR "tirofiban" OR "eptifibatide" OR "dipyridamole" OR "picotamide" OR "ticlopidine" OR "indobufen" OR "iloprost" OR "triflusal" OR "cilostazol" OR "vorapaxar" OR "heparin" OR "antithrombin III" OR "dalteparin" OR "enoxaparin" OR "nadroparin" OR "danaparoid" OR "fondaparinux" OR "parnaparin" OR "reviparin" OR "tinzaparin" OR "sulodexide" OR "bemiparin" OR "warfarin" OR "fenprocoumon" OR "acenocoumarol" OR "dicoumarol" OR "phenindione" OR "fluindione" OR "dabigatran" OR "desirudin" OR "lepirudin" OR "argatroban" OR "melagatran" OR "ximelagatran" OR "bivalirudin" OR "rivaroxaban" OR "apixaban" OR "edoxaban" OR "betrixaban"):TI,AB,KY |
| **Patient populations** | |
| #2 | ((((Cardiovascular or coronary or heart) adj3 diseas*) OR (Angina adj pectoris) OR (Atrial adj fibrillation) OR (Heart adj failure) OR (Myocardial adj (infarction or ischaemia)) OR (Acute-Coronary adj Syndrome)) OR ((Aortic adj aneurysm) OR (Brain adj (infarction or ischemia)) OR (Cerebrovascular adj (disorder* or accident)) OR ((Peripheral or vascular) adj2 disease) OR Stroke) OR ((Diabetes adj Mellitus) OR Hypertension OR Dyslipidemia* OR Hypercholesterolemia OR Atherosclerosis OR (Metabolic adj Syndrome) OR (chronic adj renal-insufficiency) or (chronic adj Kidney-Failure) OR (end-stage adj kidney-disease))):TI,AB,KY |
| **Clinical trial filter** | |
| #3 | clinicaltrial*:TR |
| **Combine interventions, population and trial filter** | |
| #4 | #1 AND #2 AND #3 |
| **Add NOT terms** | |
| #5 | (pharmacokinetic* OR pharmacology OR pharmacodynamic* OR 'drug interaction' OR drug-interaction OR 'pharmacokinetic interaction' OR biomarker OR neoplas* OR tumor OR tumour OR cancer OR malignant OR malignancy OR malignancies OR 'hematologic neoplasm' OR 'haematologic neoplasm' OR 'haematological malignancy' OR 'hematological malignancy' OR 'leukaemia' OR 'leukemia' OR lymphoma OR 'chronic obstructive pulmonary disease' OR COPD OR 'pulmonary hypertension' OR 'asthma' OR 'ablation techniques' OR 'ambulatory surgical procedures' OR (anastomosis adj Roux-en-Y) OR 'Blalock-Taussig Procedure' OR 'Cerebrospinal fluid shunts' OR cholecystectomy OR choledochostomy OR 'endolymphatic shunt' OR gastroenterostomy OR 'jejunoileal bypass' OR pancreatojejunostomy OR 'pericardial window techniques' OR 'peritoneovenous shunt' OR (portoenterostomy adj2 hepatic) OR 'salpingostomy' OR Vasovasostomy OR 'anterior temporal lobectomy' OR 'bariatric surgery' OR 'cytoreduction surgical procedures' OR 'debridement' OR (Decompression adj surgical) OR Dissection OR drainage OR 'elective surgical procedures' OR electrosurgery OR 'endocrine surgical procedures' OR 'fasciotomy' OR (Hemostasis adj2 surgical) OR Keratectomy OR laparotomy OR 'lymph node excision' OR mastectomy OR metastasectomy OR 'minimally invasive surgical procedures' OR 'minor surgical procedures' OR myotomy OR 'neurosurgical procedures' OR 'obstetric surgical procedures' OR 'ophthalmologic surgical procedures' OR 'Oral surgical procedures' OR 'orthopedic procedures' OR 'orthopaedic procedures' OR ostomy OR 'Otorhinolaryngologic Surgical Procedures' OR 'perioperative care' OR 'perioperative period' OR 'pneumonectomy' OR 'prophylactic surgical procedures' OR 'arthroplasty, replacement' OR 'auditory brain stem implantation' OR 'breast implantation' OR 'Cochlear implantation' OR 'dental implantation' OR 'maxillofacial prosthesis implantation' OR 'ossicular replacement' OR 'penile implantation' OR 'reconstructive surgical procedures' OR reoperation OR 'second-look surgery' OR 'splenectomy' OR 'surgery, computer-assisted' OR 'Symphysiotomy' OR 'Mediastinoscopy' OR 'Pulmonary surgical procedures' OR sternotomy OR thoracoplasty OR thoracoscopy OR thoracostomy OR thoracotomy OR thymectomy OR tracheostomy OR tracheotomy OR transplantation OR 'Ultrasonic surgical procedures' OR 'urogenital surgical procedures' OR 'wound closure techniques' OR 'paediatric' OR pediatric OR child OR children OR adolescent OR neonate OR newborn OR baby OR 'Pre-Eclampsia' OR 'HELLP Syndrome' OR (Hypertension-Pregnancy adj Induced) OR (Delivery adj Obstetric) OR Eclampsia OR Pregnancy OR pre-eclampsia OR 'HELLP syndrome' OR Hemolysis or (Elevated-Liver adj enzymes) or Low-Platelets OR 'pregnancy-induced hypertension' OR 'gestational hypertension' OR 'eclampsia'):TI,AB,KY |
| **Combine NOT terms with population, interventions, and time filter** | |
| #6 | #4 NOT #5 |

**Appendix 2: search strategy for validation study**

| **Included interventions** | |
| --- | --- |
| #1 – platelet inhibitors + anticoagulants | "Aspirin"[Mesh] OR aspirin [tiab] OR "acetylsalicylic acid"[tiab] OR "Carbasalate calcium" [tiab] OR clopidogrel [mesh] OR clopidogrel [tiab] OR prasugrel [mesh] OR prasugrel [tiab] OR ticagrelor [mesh] OR ticagrelor [tiab] OR cangrelor [tiab] OR abciximab [mesh] OR abciximab [tiab] OR tirofiban [mesh] OR tirofiban [tiab] OR eptifibatide [mesh] OR eptifibatide [tiab] OR dipyridamole [mesh] OR dipyridamole [tiab] OR picotamide [tiab] OR ticlopidine [mesh] OR ticlopidine [tiab] OR iloprost [mesh] OR iloprost [tiab] OR triflusal [tiab] OR cilostazol [mesh] OR cilostazol [tiab] OR vorapaxar [tiab] OR heparin [mesh] OR heparin [tiab] OR "antithrombin III" [mesh] OR "antithrombin III" [tiab] OR dalteparin [mesh] OR dalteparin [tiab] OR enoxaparin [mesh] OR enoxaparin [tiab] OR nadroparin [mesh] OR nadroparin [tiab] OR danaparoid [tiab] OR fondaparinux [mesh] OR fondaparinux [tiab] OR parnaparin [tiab] OR reviparin [tiab] OR tinzaparin [mesh] OR tinzaparin [tiab] OR sulodexide [tiab] OR bemiparin [tiab] OR warfarin [mesh] OR warfarin [tiab] OR phenprocoumon [mesh] OR phenprocoumon [tiab] OR acenocoumarol [mesh] OR acenocoumarol [tiab] OR dicoumarol [mesh] OR dicoumarol [tiab] OR phenindione [mesh] OR phenindione [tiab] OR fluindione [tiab] OR dabigatran [mesh] OR dabigatran [tiab] OR desirudin [tiab] OR lepirudin [tiab] OR argatroban [tiab] OR melagatran [tiab] OR ximelagatran [tiab] OR bivalirudin [tiab] OR rivaroxaban [mesh] OR rivaroxaban [tiab] OR  apixaban [tiab] OR edoxaban [tiab] OR betrixaban [tiab] |
| **Patient population** | |
| #2 – cardiovascular disease | "cardiovascular diseases"[mesh] OR "cardiovascular disease" [tiab] OR "heart diseases"[mesh] OR "heart disease" [tiab] OR “coronary artery disease”[mesh] OR “coronary artery disease”[tiab] OR "coronary heart disease" [tiab] OR "Percutaneous Coronary Intervention"[Mesh] OR "Angioplasty, Balloon, Coronary"[Mesh] OR "percutaneous coronary intervention"[tiab] OR PCI [tiab] OR “percutaneous transluminal coronary angioplasty”[tiab] OR “coronary revascularisation”[tiab] OR “coronary angioplasty”[tiab] OR “coronary stent*”[tiab] OR "Coronary Artery Bypass"[Mesh] OR "coronary artery bypass" [tiab] OR "coronary artery bypass graft*" [tiab] OR CABG [tiab] OR “coronary bypass surgery”[tiab] OR "Myocardial Infarction"[Mesh] OR "ST Elevation Myocardial Infarction"[Mesh] OR "Non-ST Elevated Myocardial Infarction"[Mesh] OR "Myocardial Ischemia"[Mesh] OR “myocardial infarction”[tiab] OR “ST elevation myocardial infarction”[tiab] OR STEMI [tiab] OR “non-st elevation myocardial infarction”[tiab] OR “non-stemi” [tiab] OR “myocardial ischaemia” [tiab] OR “myocardial ischemia”[tiab] OR “heart attack”[tiab] OR "Atrial Fibrillation"[Mesh] OR "Arrhythmias, Cardiac"[Mesh] OR “atrial fibrillation”[tiab] OR “cardiac arrhythmia*”[tiab] OR "Heart Failure"[Mesh] OR "Heart Failure, diastolic"[Mesh] OR "Heart Failure, systolic"[Mesh] OR “Heart failure”[tiab] OR “Heart decompensation”[tiab] OR “cardiac decompensation”[tiab] OR “myocardial failure”[tiab] OR “right-sided heart failure”[tiab] OR “left-sided heart failure”[tiab] OR “congestive heart failure”[tiab] OR “cardiac insufficiency”[tiab] OR “systolic heart failure”[tiab] OR “diastolic heart failure”[tiab] OR “heart failure with preserved ejection fraction”[tiab] OR HFREF[tiab] OR “HF-REF”[tiab] OR “heart failure with reduced ejection fraction”[tiab] OR HFPEF[tiab] OR “HF-PEF”[tiab] |
| #3 vascular disease | "Vascular Diseases"[Mesh] OR "Peripheral Vascular Diseases"[Mesh] OR "Peripheral Arterial Disease"[Mesh] OR “diabetic foot”[mesh] OR "Angioplasty"[Mesh] OR "Intermittent Claudication"[Mesh] OR "Amputation"[Mesh] OR “vascular disease”[tiab] OR “peripheral vascular disease”[tiab] OR “peripheral arterial disease”[tiab] OR PAD[tiab] OR “diabetic foot”[tiab] OR angioplasty[tiab] OR “bypass graft”[tiab] OR “peripheral bypass graft”[tiab] OR “peripheral angioplasty”[tiab] OR “peripheral revascularisation”[tiab] OR “amputation”[tiab] OR "Aortic Aneurysm"[Mesh] OR “Aortic Aneurysm, abdominal”[mesh] OR “aortic aneurysm”[tiab] OR “abdominal aortic aneurysm”[tiab] OR AAA[tiab] OR "Stroke"[Mesh] OR “Cerebrovascular disorders”[mesh] OR "Ischemic Attack, Transient"[Mesh] OR "Brain Ischemia"[Mesh] OR "Cerebral Infarction"[Mesh] OR "Cerebral Hemorrhage"[Mesh] OR "Ischemic Stroke"[Mesh] OR "Hemorrhagic Stroke"[Mesh] OR stroke [tiab] OR “cerebrovascular disease”[tiab] OR “cerebrovascular disorder*”[tiab] OR “transient ischaemic attack”[tiab] OR “transient ischemic attack”[tiab] OR TIA[tiab] OR “brain ischaemia”[tiab] OR “brain ischemia”[tiab] OR “cerebral infarction”[tiab] OR “cerebral haemorrhage”[tiab] OR “cerebral hemorrhage”[tiab] OR “ischaemic stroke”[tiab] OR “ischemic stroke”[tiab] OR “haemorrhagic stroke”[tiab] OR “hemorrhagic stroke”[tiab] OR CVA[tiab] |
| #4 – CVD risk factors | "Heart Disease Risk Factors"[Mesh] OR "Metabolic Syndrome"[Mesh] OR "Hypertension"[Mesh] OR “essential hypertension”[mesh] OR "Prediabetic State"[Mesh] OR "Diabetes Mellitus"[Mesh] OR "Diabetes Mellitus, Type 1"[Mesh] OR "Diabetes Mellitus, Type 2"[Mesh] OR "Hyperglycemia"[Mesh] OR "Overweight"[Mesh] OR "Obesity"[Mesh] OR "Hyperlipidemias"[Mesh] OR "Dyslipidemias"[Mesh] OR "Renal Insufficiency"[Mesh] OR "Kidney Failure, Chronic"[Mesh] OR "Renal Dialysis"[Mesh] OR “cardiovascular risk factor*”[tiab] OR “cardiometabolic risk factor*”[tiab] OR “cardiovascular risk”[tiab] OR “metabolic syndrome”[tiab] OR “cardiometabolic syndrome”[tiab] OR hypertension [tiab] OR “arterial hypertension”[tiab] OR “essential hypertension”[tiab] OR “high blood pressure”[tiab] OR diabetes [tiab] OR “diabetes mellitus”[tiab] OR “pre-diabetes”[tiab] OR “type 1 diabetes”[tiab] OR “diabetes type 1”[tiab] OR “type I diabetes”[tiab] OR “diabetes type I”[tiab] OR “type 2 diabetes”[tiab] OR “diabetes type 2”[tiab] OR “type II diabetes”[tiab] OR “diabetes type II”[tiab] OR hyperglycaemia[tiab] OR hyperglycemia[tiab] OR dysglycaemia[tiab] OR dysglycemia[tiab] OR “insulin resistance”[tiab] OR “glucose intolerance”[tiab] OR overweight[tiab] OR “obesity”[tiab] OR obese[tiab] OR “morbid obesity”[tiab] OR “morbidly obese”[tiab] OR hyperlipidaemia[tiab] OR hyperlipidemia[tiab] OR hypercholesterolaemia[tiab] OR hypercholesterolemia[tiab] OR dyslipidaemia[tiab] OR dyslipidemia[tiab] OR “familial hypercholesterolaemia”[tiab] OR “familial hypercholesterolemia”[tiab] OR “familial hyperlipidaemia”[tiab] OR “familial hyperlipidemia”[tiab] OR “chronic kidney disease”[tiab] OR CKD[tiab] OR “chronic renal disease”[tiab] OR “kidney failure”[tiab] OR “renal failure”[tiab] OR “kidney insufficiency”[tiab] OR “renal insufficiency”[tiab] OR “kidney disfunction”[tiab] OR “renal disfunction”[tiab] OR “end-stage kidney disease”[tiab] OR “end-stage renal disease”[tiab] OR ESKD[tiab] OR ESRD[tiab] OR dialysis[tiab] OR “kidney dialysis”[tiab] OR “renal dialysis”[tiab] OR “haemodialysis”[tiab] OR hemodialysis[tiab] OR “maintenance dialysis”[tiab] OR “maintenance haemodialysis”[tiab] OR “maintenance hemodialysis”[tiab] OR “peritoneal dialysis” OR HD[tiab] OR PD[tiab] |
| #5 – combine CVD, PVD, and CVD risk factors | #2 OR #3 OR #4 |
| **Combine population with interventions** | |
| #6 | #1 AND #5 |
| **Apply time filter** | |
| #7 | #6 + time filter ≥2012 |
| **Add NOT terms** | |
| #8 – pharmacological | "Pharmacokinetics"[Mesh] OR "Pharmacology"[Mesh] OR pharmacokinetic* [tiab] OR pharmacology[tiab] OR pharmacodynamic*[tiab] OR “drug interaction”[tiab] OR “drug-interaction”[tiab] OR “pharmacokinetic interaction”[tiab] |
| #9 – biomarker | "Biomarkers"[Mesh] OR biomarker[tiab] |
| #10 - oncology | neoplasms[Mesh] OR "Hematologic Neoplasms"[Mesh] OR “neoplas*”[tiab] OR tumor[tiab] OR tumour[tiab] OR cancer[tiab] OR malignant[tiab] OR malignancy[tiab] OR malignancies[tiab] OR “hematologic neoplasm”[tiab] OR “haematologic neoplasm”[tiab] OR “haematological malignancy”[tiab] OR “hematological malignancy”[tiab] OR “leukaemia”[tiab] OR “leukemia”[tiab] OR lymphoma[tiab] |
| #11 - lung disease | "Pulmonary Disease, Chronic Obstructive"[Mesh] OR "Asthma"[Mesh] OR "Hypertension, Pulmonary"[Mesh] OR “chronic obstructive pulmonary disease”[tiab] OR COPD[tiab] OR “pulmonary hypertension”[tiab] OR “asthma”[tiab] |
| #12 - surgery | “ablation techniques”[Mesh] OR “ambulatory surgical procedures”[Mesh] OR “anastomosis, Roux-en-Y”[Mesh] OR “Blalock-Taussig Procedure”[Mesh] OR “Cerebrospinal fluid shunts”[Mesh] OR cholecystectomy[Mesh] OR choledochostomy[Mesh] OR “endolymphatic shunt”[Mesh] OR gastroenterostomy[Mesh] OR “jejunoileal bypass”[Mesh] OR pancreatojejunostomy[Mesh] OR “pericardial window techniques”[Mesh] OR “peritoneovenous shunt”[Mesh] OR “portoenterostomy, hepatic”[Mesh] OR “salpingostomy”[Mesh] OR Vasovasostomy[Mesh] OR “anterior temporal lobectomy”[Mesh] OR “bariatric surgery”[Mesh] OR “cytoreduction surgical procedures” OR “debridement”[Mesh] OR “Decompression, surgical”[Mesh] OR Dissection[Mesh] OR drainage[Mesh] OR “elective surgical procedures”[Mesh] OR electrosurgery[Mesh] OR “endocrine surgical procedures”[Mesh] OR “fasciotomy”[Mesh] OR “Hemostasis, surgical”[Mesh] OR Keratectomy[Mesh] OR laparotomy[Mesh] OR “lymph node excision”[Mesh] OR mastectomy[Mesh] OR metastasectomy[Mesh] OR “minimally invasive surgical procedures”[Mesh] OR “minor surgical procedures”[Mesh] OR myotomy[Mesh] OR “neurosurgical procedures”[Mesh] OR “obstetric surgical procedures”[Mesh] OR “ophthalmologic surgical procedures”[Mesh] OR “Oral surgical procedures”[Mesh] OR “orthopedic procedures”[Mesh] OR ostomy[Mesh] OR “Otorhinolaryngologic Surgical Procedures”[Mesh] OR “perioperative care”[Mesh] OR “perioperative period”[Mesh] OR “pneumonectomy”[Mesh] OR “prophylactic surgical procedures”[Mesh] OR “arthroplasty, replacement”[Mesh] OR “auditory brain stem implantation”[Mesh] OR “breast implantation”[Mesh] OR “Cochlear implantation”[Mesh] OR “dental implantation”[Mesh] OR “maxillofacial prosthesis implantation”[Mesh] OR “ossicular replacement”[Mesh] OR “penile implantation”[Mesh] OR “reconstructive surgical procedures”[Mesh] OR reoperation[Mesh] OR “second-look surgery”[Mesh] OR “splenectomy”[Mesh] OR “surgery, computer-assisted”[Mesh] OR “Symphysiotomy”[Mesh] OR “Mediastinoscopy”[Mesh] OR “Pulmonary surgical procedures”[Mesh] OR sternotomy[Mesh] OR thoracoplasty[Mesh] OR thoracoscopy[Mesh] OR thoracostomy[Mesh] OR thoracotomy[Mesh] OR thymectomy[Mesh] OR tracheostomy[Mesh] OR tracheotomy[Mesh] OR transplantation[Mesh] OR “Ultrasonic surgical procedures”[Mesh] OR “urogenital surgical procedures”[Mesh] OR “wound closure techniques”[Mesh] |
| #13 - paediatric | “child”[Mesh] OR "Adolescent"[Mesh] OR "Infant, Newborn"[Mesh] OR “paediatric”[tiab] OR pediatric[tiab] OR child[tiab] OR children[tiab] OR adolescent[tiab] OR neonate[tiab] OR newborn[tiab] OR baby[tiab] |
| #14 – pregnancy | "Pre-Eclampsia"[Mesh] OR "HELLP Syndrome"[Mesh] OR "Hypertension, Pregnancy-Induced"[Mesh] OR "Delivery, Obstetric"[Mesh] OR "Eclampsia"[Mesh] OR "Pregnancy"[Mesh] OR “pre-eclampsia”[tiab] OR “HELLP syndrome”[tiab] OR “Hemolysis, Elevated Liver enzymes and Low Platelets”[tiab] OR “Haemolysis, Elevated Liver enzymes and Low Platelets”[tiab] OR “pregnancy induced hypertension”[tiab] OR “pregnancy-induced hypertension”[tiab] OR “gestational hypertension”[tiab] OR “eclampsia”[tiab] |
| **Combine NOT terms with population, interventions, and time filter** | |
| #15 | #7 NOT (#8 OR #9 OR #10 OR #11 OR #12 OR #13 OR #14) |
